# Is parental socioeconomic status associated with irregularity of energy intake among children?

**DOI:** 10.1093/eurpub/ckac131.232

**Published:** 2022-10-25

**Authors:** E Roos, R Pajulahti, R Lehto, K Nissinen, M Erkkola, C Ray, L Korkalo

**Affiliations:** 1 Folkhälsan Research Center, Samfundet Folkhälsan, Helsinki, Finland; 2 Department of Food Studies, Nutrition and Diet, Uppsala University, Uppsala, Sweden; 3 Department of Food and Nutrition, University of Helsinki, Helsinki, Finland

## Abstract

**Background:**

The timing of eating, chrono-nutrition, is a relatively new research area, where the focus is not on only what we eat but also when we eat and of irregularity between days. Chrono-nutrition have been associated with cardiovascular risk factors among adults. Societal factors influence the chrono-nutrition, but there is limited research on how different socioeconomic factors are associated with chrono-nutrition, especially among children. The aim of this study is to examine the association between parental socioeconomic status and irregularity of children’s energy intake.

**Methods:**

We used data from the DAGIS (Increased Health and Wellbeing in Preschools) study in years 2015-2016, in which 864 preschool children participated (age 3-6 years old). Childreńs dietary intake was measured by a 3-day food record. We included only children that had complete data from all three days and the child was at day care on two of those days and one day was a weekend day (n = 568). To calculate the irregularity score, the absolute difference between the daily energy intake and 3-day mean intake for each three days was divided by the 3-day mean energy intake, multiplied by 100 and then averaged over the 3 days; this served as a measure of irregularity of energy intake, with a low score indicating a more regular energy intake and a higher more irregular energy intake. The parents reported highest parental educational level and household income on a questionnaire. The associations were tested by general linear models on SPSS.

**Results:**

No association was found between parental educational level or household income with irregularity of children’s energy intake.

**Conclusions:**

There was no association between parental socioeconomic status and children’s irregularity of energy intake. Other aspects of children’s chrono-nutrition should also be tested to be able to conclude that children’s chrono-nutrition is not influenced by parental socioeconomic status.

**Key messages:**

• We found no association between parental socioeconomic status and irregularity in children’s energy intake.

• Chrono-nutrition among children is under-examined.

